# Cholinergic modulation of hippocampal activity during septic encephalopathy

**DOI:** 10.1186/cc11962

**Published:** 2013-03-19

**Authors:** A Zivkovic, CP Bengtson, O Sedlaczek, R Von Haken, H Bading, S Hofer

**Affiliations:** 1 Universitätsklinikum Heidelberg, Germany; 2 Universität Heidelberg, Germany

## Introduction

Septic encephalopathy is a sepsis-related brain dysfunction with a deterioration of cortical functions. The experimental studies in the rat brain revealed a deranged neurotransmitter proflle during septic encephalopathy. Glutamatergic synapses, essential in learning and memory, undergo use-dependent changes in synaptic strength, referred to as plasticity. Permanent strengthening of synapses after a brief stimulus, termed long-term potentiation (LTP), was discovered in the hippocampus, and here it has been most thoroughly studied. Cholinergic neurotransmission plays an important role in regulating the cognitive functions of the brain. It acts as a signal-to-noise ratio modulator of sensory and cognitive inputs. The irregularities in brain functions give rise to the symptoms of delirium, including disorganized thinking and disturbances of attention and consciousness, which in turn might affect learning and memory. Possible mechanisms for cholinergic deficiency include impairment of synaptic functions of acetylcholine. Imbalances in the cholinergic system during sepsis might therefore play an extensive role in the septic delirium.

## Methods

By using MRI imaging we identified functional changes in the hippocampal region of patients with severe sepsis. This finding was further supported by the experimental recordings in the rat brains of lipopolysaccharide (LPS)-treated rats using the electrophysiological patch clamp technique.

## Results

Critically ill ICU patients diagnosed with septic delirium using the CAM-ICU method underwent diagnostic MRI scans. The serial MRI analysis revealed increased signal intensity in the hippocampal region in diffusion-weighted MRI (DWI). We used endotoxemia model to induce sepsis in the rats. Electrophysiological analysis of the hippocampal neurons in LPS-treated rats showed impaired LTP in the excitatory synapses, as compared with controls. Application of physostigmine, a blood-brain barrier permeable cholinesterase inhibitor, resulted in a partial recovery of LTP in the hippocampal synapses of LPS-treated rats.

## Conclusion

The patients with septic delirium show functional changes in the hippocampus. Furthermore, we show that endotoxemia affects synaptic plasticity in the rat hippocampus, suggesting the involvement of this brain region in the pathophysiology of septic delirium. Moreover, the effect of the cholinergic neurotransmission onto the induction and maintenance of synaptic plasticity in the rat hippocampus during endotoxemia suggests that cholinergic neurotransmission might play a critical role in septic encephalopathy.

